# Perceptual impressions of causality are affected by common fate

**DOI:** 10.1007/s00426-017-0853-y

**Published:** 2017-03-24

**Authors:** Peter A. White

**Affiliations:** School of Psychology, Cardiff University, Tower Building, Park Place, Cardiff, Wales CF10 3YG UK

## Abstract

Many studies of perceptual impressions of causality have used a stimulus in which a moving object (the launcher) contacts a stationary object (the target) and the latter then moves off. Such stimuli give rise to an impression that the launcher makes the target move. In the present experiments, instead of a single target object, an array of four vertically aligned objects was used. The launcher contacted none of them, but stopped at a point between the two central objects. The four objects then moved with similar motion properties, exhibiting the Gestalt property of common fate. Strong impressions of causality were reported for this stimulus. It is argued that the array of four objects was perceived, by the likelihood principle, as a single object with some parts unseen, that the launcher was perceived as contacting one of the unseen parts of this object, and that the causal impression resulted from that. Supporting that argument, stimuli in which kinematic features were manipulated so as to weaken or eliminate common fate yielded weaker impressions of causality.

## Introduction

It is well established that certain configurations of moving objects give rise to perceptual impressions of causality (Hubbard 2013a, b; Michotte 1963; Scholl and Tremoulet 2000). The two best known examples of perceptual impressions of causality, also known as “phenomenal causality” (Michotte 1963), are the launching and entraining effects. In the typical stimulus for launching, a stationary object (the target) is visible near the centre of the screen. A moving object (the launcher) enters from the side, moving horizontally on a path that takes it to contact with the target. When the launcher contacts the target, the target stops moving and the launcher starts moving in the same direction and with the same or slightly lesser speed. Observers consistently report an impression that the launcher made the target move by bumping into it (Hubbard 2013a, b; Michotte 1963; Scholl and Tremoulet 2000). The typical stimulus for entraining is similar except that the object perceived as causal in this case, called the entrainer, continues to move after contacting the target, and the two objects remain in contact. Observers consistently report an impression that the entrainer pushes or carries the target (Hubbard 2013a, b; Michotte 1963; Scholl and Tremoulet 2000).

Michotte (1963) proposed explanations for these causal impressions that owed much to principles of perceptual grouping in Gestalt psychology. In particular, in the case of entraining, Michotte proposed that the impression was mediated by the principle of common fate. Common fate is perceptual grouping of objects by possession of similar motion properties (see Wagemans et al. 2012, for a recent review). For example, a number of spatially separated geometrical objects moving in a uniform way across a field will tend to pop out of a background as a result of grouping by common fate (Wertheimer 1923). Thus, the common motion properties of entrainer and target after contact counted as an example of common fate, integrating the two objects in a perceptual group, resulting in a unified percept in which the common motion of the two objects is that of the entrainer (Michotte 1963).

Michotte’s interpretation of entraining is not universally accepted and several other hypotheses have been proposed (Hubbard 2013a, b; Scholl and Tremoulet 2000; White (in press)). In this paper, however, I argue that common fate has a different kind of relevance to both the launching and entraining effects. Several studies have shown that the launching effect is absent or greatly weakened if there is a gap between the two objects; that is, if the launcher stops moving before it contacts the target (Michotte 1963; Yela 1952; Young and Falmier 2008). I test the hypothesis that a stronger causal impression can be obtained for both launching and entraining stimuli if the launcher or entrainer stops at a location perceptually interpreted as part of a perceptual object created by perceptual grouping of visible objects according to common fate.

**Fig. 1:**
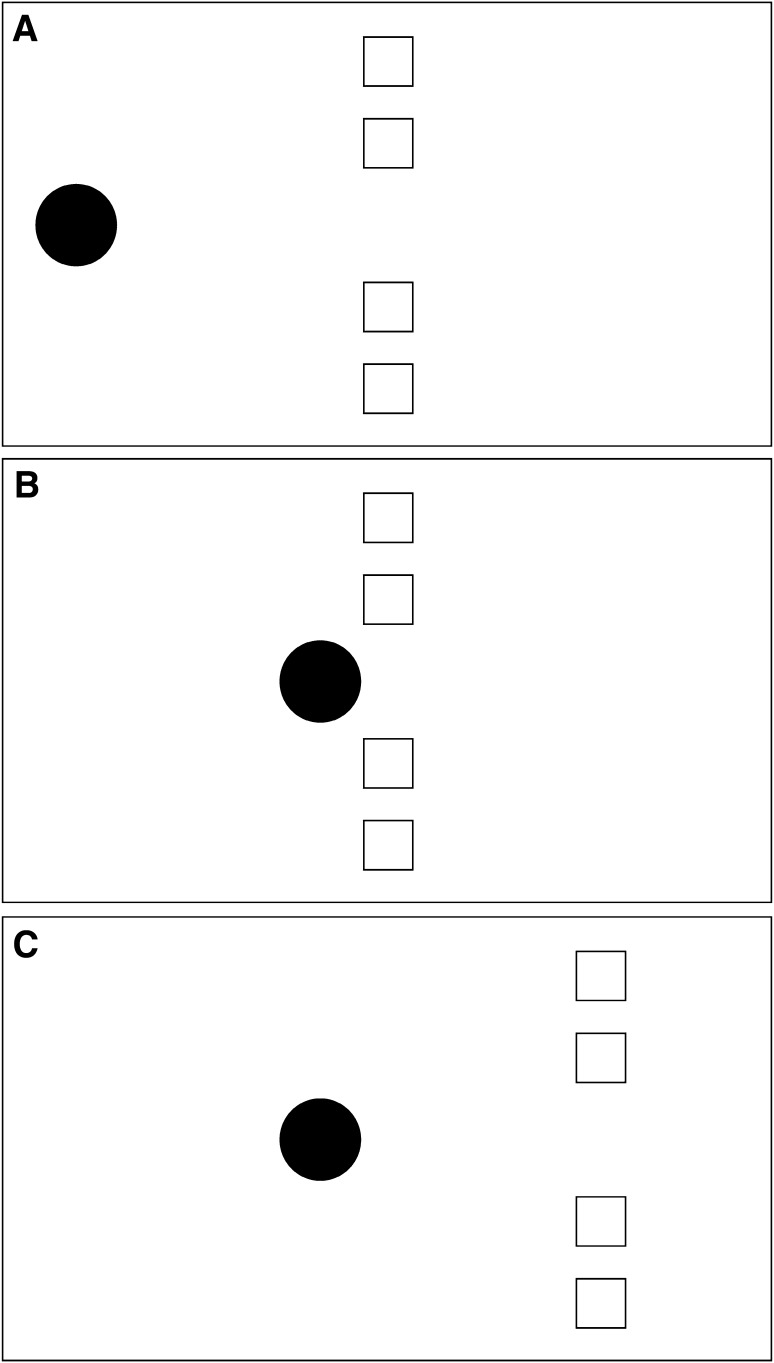
Schematic representation of the launching version of the common fate stimulus with a single object as launcher. **A** shows the first frame of the stimulus. The four *white squares* near the centre of the frame are the target and the *black disc* is the launcher. The launcher moves horizontally to the *right*. **B** shows the frame in which the launcher stops moving. Without delay, the target objects start moving horizontally to the *right*. **C** shows a frame from that phase of the stimulus. Eventually the target objects exit the frame at the *right-hand side*

The basic stimulus for launching in this study is shown in Fig. 1. The first part of this figure shows the first frame of the stimulus, with four objects in a vertical arrangement with gaps between them. These are the target objects and they are initially stationary. The launcher is visible at the left side of the frame. The launcher moves horizontally across the frame at a constant speed. The second part of Fig. 1 shows the location at which the launcher stops moving. It has not contacted any of the four target objects, but it has stopped at a point that would be considered as contacting an object if there was in fact a single object with the breadth of the target objects extending down the screen from the highest target to the lowest. At that moment, the four target objects start moving horizontally to the right at constant speed. The third part of Fig. 1 shows a later stage in this motion, which continues until the objects exit the screen.

It is hypothesized that the initial location and subsequent motion of the four target objects, conforming to the common fate principle, perceptually establishes them as the visible parts of a single object extending vertically across the space the objects delimit. This is essentially an application of the likelihood principle, first formulated by Helmholtz (Wagemans et al. 2012). This principle states that “the perceptual system determines the most likely distal stimulus that could have given rise to the proximal stimulus (the retinal image)” (Wagemans et al. 2012, p. 1225). Thus, it is extremely unlikely that four separate objects would share precisely the same motion properties, including both onset time and velocity, but rather less unlikely that they are in fact the visible parts of a single object. The object might, for example, be partly occluded by foreground objects.

**Fig. 2:**
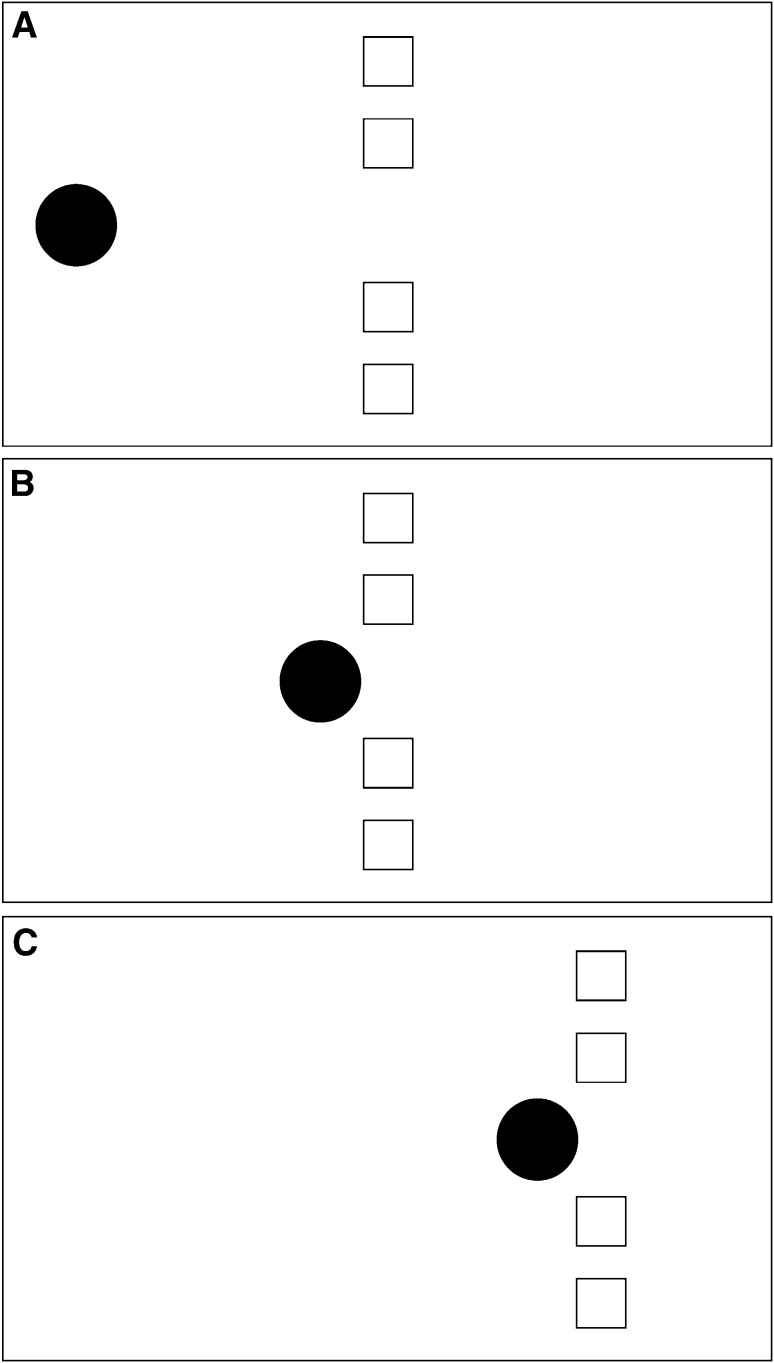
Schematic representation of the entraining version of the common fate stimulus with a single object as entrainer. Figure 1A shows the first frame of the stimulus. The four *white squares* near the centre of the frame are the target and the *black disc* is the entrainer. The stimulus is identical to the launching stimulus shown in Fig. 1, except that, when the *black disc* reaches the point where the launcher stops, it continues moving at the same speed as the targets: this is depicted in Fig. 1C

Under that interpretation, the launcher does not stop at a distance from the target objects, but is perceived as directly contacting the unseen boundary of an object of which the targets are the visible parts. Therefore, a relatively strong impression of launching should occur with this stimulus. The word “relatively” is used with the implication of suitable control stimuli, which will be described later. In Experiment 1, an entraining version of this stimulus was used as well. This is depicted schematically in Fig. 2. As the figure shows, the stimulus is similar to the launching stimulus except that the entrainer continues moving with the same motion properties as the target objects. A relatively strong impression of entraining should occur with this stimulus.

**Fig. 3:**
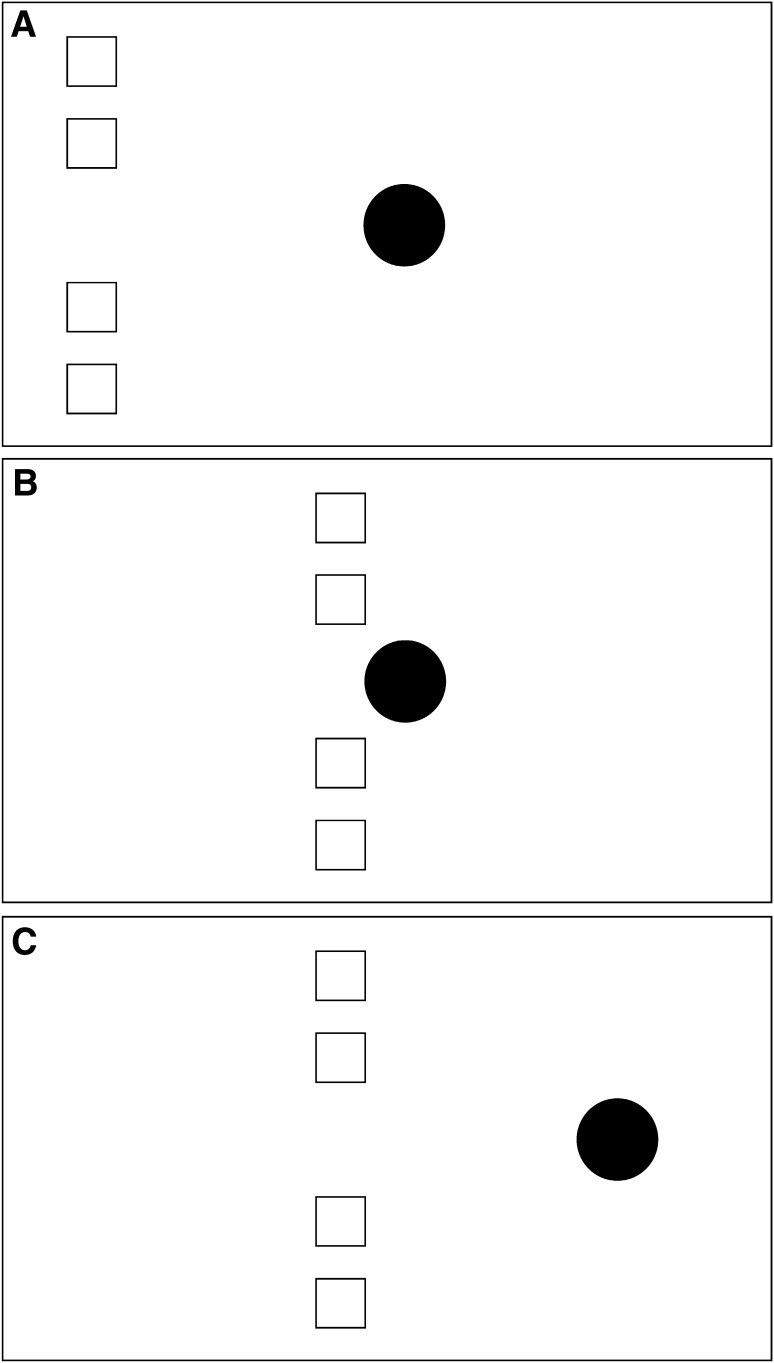
Schematic representation of the launching version of the common fate stimulus with multiple objects as launcher. The motion properties are exactly as in the Fig. 1 stimulus, but the roles of the four *white squares* and the *black disc* are reversed, as shown

**Fig. 4:**
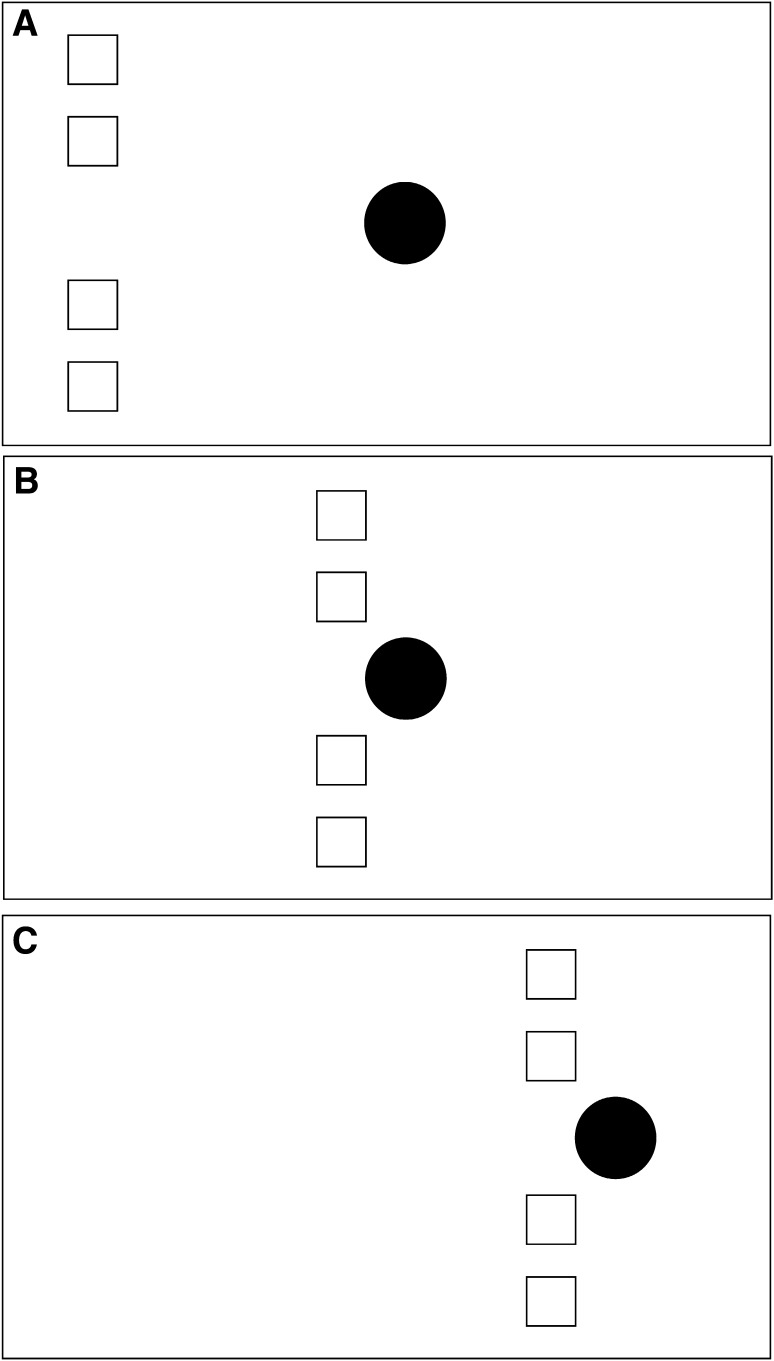
Schematic representation of the entraining version of the common fate stimulus with multiple objects as launcher. The motion properties are exactly as in the Fig. 2 stimulus, but the roles of the four *white squares* and the *black disc* are reversed, as shown

In the stimuli depicted in Figs. 1 and 2, the launcher/entrainer is a single object and the target comprises multiple objects. This can of course be reversed so that the launcher/entrainer comprises multiple objects and the target is a single object. Since the motion of the launcher/entrainer establishes common fate for the four objects, the causal impression should be just as strong for this kind of stimulus as for the Figs. 1 and 2 stimuli. These versions of the stimulus are depicted in Fig. 3 (launching) and Fig. 4 (entraining).

The basic hypothesis is that the strength of the causal impression should be affected by factors that affect the occurrence and strength of perceptual grouping by common fate. Two experiments are reported that test the predicted effects of such manipulations. For ease of reference, the independent variables in the two experiments are listed in Table 1.

**Table 1:**
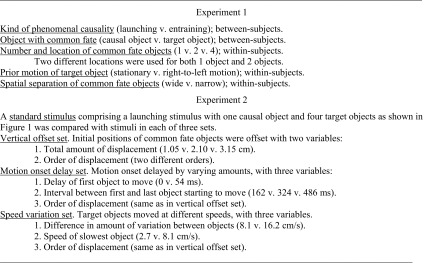
Summary of the manipulations in the two experiments

## Experiment 1

The main aim of Experiment 1 was to test the hypothesis that stronger causal impressions would occur for stimuli where perceptual grouping of the targets by common fate is strong than for stimuli where it is weak or absent. Some conditions affecting perceptual grouping by common fate have been elucidated in studies by Uttal, Spillmann, Stürzel, and Sekuler (2000) and Stürzel and Spillmann (2004). In those studies, observers were required to detect common fate shared by objects in a context of other objects with different motion properties. No such context is used in the present research, but it is still predicted that the strength of the causal impression should vary in a manner predictable from the findings of the detection studies.

Where there is only one target object there is no common fate, so a stimulus presenting just one of the target objects shown in Figs. 1 and 2 should generate a weaker causal impression than that generated by the Figs. 1 and 2 stimuli. Where there are two target objects, common fate can occur if they share motion properties. Likelihood of detection of the target stimulus increases as the number of objects increases up to about four (Stürzel and Spillmann 2004; Uttal et al. 2000). This implies that perceptual grouping by common fate is weaker with two objects than with four. This therefore predicts that versions of the Figs. 1 and 2 stimuli with just two of the four target objects visible should yield weaker causal impressions than the Figs. 1 and 2 stimuli, but the causal impressions should still be stronger than for the stimuli with just one visible target object.

Uttal et al. (2000) and Stürzel and Spillmann (2004) also found an approximately linear relation between spacing of the objects and likelihood of detection, with greater likelihood of detection at close spacing. This generates the prediction that the causal impression should be stronger if the target objects are closely spaced than if they are spaced further apart.

Finally, the target in Figs. 1, 2, 3 and 4 is stationary until the launcher or entrainer stops moving. This means that perceptual grouping by common fate is not established until after the causal object has stopped moving. In Experiment 1, each stimulus was compared with one in which the target was initially positioned at the right side of the frame and moved towards the centre. The motion was timed so that the target reached the position shown in panel B of each of the four figures at the same moment that the launcher/entrainer reached the position shown in panel B of each figure. At that moment, the target reversed direction and moved as in the four figures after contact. Thus, everything was the same except for the prior motion of the target. In the prior motion stimuli, perceptual grouping by common fate is established before the launcher or entrainer has stopped moving. It was predicted that this would result in a stronger causal impression for this prior motion stimulus than for the stimuli in Figs. 1, 2, 3 and 4. There is, however, an alternative basis for predicting a stronger causal impression for the prior motion stimulus than for the stimuli in Figs. 1, 2, 3 and 4. In a real collision event, it takes more force to reverse the direction of motion of an object that is already moving than it does to set it in motion from a stationary position. Therefore, it is possible that the causal object is perceived as exerting more force on the target with prior motion than without, and that this would lead to a stronger causal impression.

The reasoning about common fate applies to the causal object as well as to the target. With the stimuli shown in Figs. 3 and 4, a causal impression should still occur because the target is perceived as being struck by a non-visible part of a single object of which the four objects are the visible parts. Exactly the same predictions hold, therefore, for the manipulations of number of visible objects and spatial separation. The prediction for effect of prior motion is different, however. Because the causal object is in motion from the start of the stimulus, perceptual grouping by common fate has already occurred before contact with the target. Therefore, manipulating the prior motion of the target should make no difference to the strength of the causal impression, insofar as that is affected by common fate. That prediction is specific to the case where the common fate object is the causal object, and differs from the prediction, given above, for the case where the common fate object is the target, the case depicted in Fig. 1. However, if the causal object is perceived as exerting more force on the target in the prior motion stimulus than in the stimulus where the target is stationary prior to contact, there should still be a significant effect of the prior motion manipulation on the strength of the causal impression.

## Method

### Participants

The participants were 120 volunteer first-year undergraduate students with normal or corrected to normal vision, participating in return for course credit. There were 30 participants in each of the four between-subject conditions described below, and assignment to conditions was random. A target sample size of 120 was set before data collection started, and data collection continued until the target was met. No participants were excluded from data analysis. Informed consent was obtained from all individual participants included in the study.

#### Apparatus and stimulus materials

Stimuli consisted of frame sequences generated by a Macintosh G3 computer and displayed on a Mitsubishi Diamond Plus 71 16″ CRT colour monitor. The frames were presented in phase with the computer’s vertical blank signal and therefore appeared at the refresh rate of 74 Hz. Each frame was 500 pixels (18.5 cm) wide by 300 pixels (11.1 cm) high. The boundaries of this frame appeared on the screen as thin black lines (to provide a rationale for the appearance and disappearance of the objects as they entered and exited the frame). These disappeared between trials, leaving the screen uniform white. All sequences consisted of 200 frames, lasting 2.7 s. The background of each frame was uniform white throughout. Stimuli were variations on those depicted in Figs. 1, 2, 3 and 4. Object boundaries were clearly defined and object motion appeared smooth to the eye.

Features common to all stimuli included speed of the causal object (18.9 cm/s) and speed of the target (13.5 cm/s). These speeds and speed ratio were chosen because they are in the range of values that maximises the strength and likelihood of occurrence of the launching and entraining effects (Michotte 1963; Natsoulas 1961). The causal objects were 1.75 cm in diameter and the target objects were 1.05 × 1.05 cm squares.

#### Design

There were five independent variables, two between-subjects and three within-subjects.

Kind of phenomenal causality (launching versus entraining) was a between-subject manipulation. The object with common fate could be either the target object, as in Figs. 1 and 2, or the causal object, as in Figs. 3 and 4, and this too was a between-subject manipulation.

The number and location of common fate objects was a within-subjects variable with five levels. There were either one, two, or four objects. However, for both one object and two objects, there was an additional manipulation of the location of these objects. Take the Fig. 1 stimulus and number the objects 1 to 4 from top to bottom. For the two-object stimuli, the two locations used were either numbers 2 and 3 or numbers 3 and 4. For the one-object stimuli, the two locations used were either number 3 or number 4.^1^

Prior motion of the target object was manipulated within-subjects with the target either being stationary (as in Figs. 1, 2, 3, 4) or moving from right to left. In the latter case, the target was initially positioned at the right edge of the frame and moved horizontally towards the *x*-coordinate of the target shown in panel B of Figs. 1, 2, 3 and 4. Its arrival at that location coincided with the arrival of the causal object at the location shown in panel B of Figs. 1, 2, 3 and 4.

Spatial separation of the common fate objects was manipulated within-subjects. Distances between the centres of the objects were either 50 pixels between objects 1 and 2 and between objects 3 and 4 and 100 pixels between objects 2 and 3 (narrow), or 80 pixels between objects 1 and 2 and between objects 3 and 4 and 100 pixels between objects 2 and 3 (wide).

The manipulations are summarised in Table 1. They yielded a total of 20 stimuli (5 × 2 × 2) per participant.

#### Procedure

The experiment was run in a small laboratory, empty except for the equipment used for the experiment and with fluorescent lighting giving a low ambient light level. Participants were seated so that their faces were approximately 75 cm. from the screen and were permitted to adjust this distance slightly for personal comfort. The experimenter introduced the experiment by giving the participant written instructions. Those for the condition with launching stimuli and the object with common fate being the target object were as follows:

“In this experiment you will see a series of short movies. In each of these movies you will see between one and four white squares that are either stationary near the centre of the screen or moving from right to left. You will also see a black disc enter from the left and move towards the centre. At some point it will stop and the squares will move from left to right.

“Your task is to answer a question concerning the visual impression you had about what made the white square(s) move. The question is:

Do you have the impression that the black disc somehow made the white square(s) move?

“Note, first, that this question is asking about however many white squares were visible. For example, if four squares were visible, the question is asking whether you had the impression that the black disc made all four of them move. Note also that the question concerns the motion of the white squares from left to right, when and after the black disc stops moving, and does not concern any movement that might have occurred before that point. If these instructions are not clear, please ask me and I will explain further.

“The question you are asked might sound odd when the black disc and the white square(s) do not come into contact, but people often do have a visual impression that one object made one or more others move even under conditions where this could not happen in real life. Sometimes, for example, they report seeing the gap between the objects as a kind of medium through which influence is transmitted from one object to another. That is what this experiment is concerned with: the visual impression you have, in other words to what extent you see the black disc make the white square(s) move, despite what you think could be possible.”

“You should answer each question by giving me a number from 0 to 100, where 0 means that you had no visual impression that the black disc made the white square(s) move at all, and 100 means that you had a very strong visual impression that the black disc made the white square(s) move. You may choose any number between 0 and 100; the more you had the visual impression that the black disc made the white square(s) move, the higher the number you should put, up to a maximum of 100. For each movie, please write down your rating in the appropriate place on the rating sheet.

“When you’ve finished reading this, I will give you further instructions on how to go through the movies.”

The written instructions for the other conditions were identical except for changes in the descriptions of the objects in accordance with differences in the stimuli. For example, where the above instructions say that the participants will see between one and four squares either stationary near the centre of the screen or moving from right to left, in conditions where the target object was a single object, this was changed to saying that the participants would see one black disc. Equivalent changes were made elsewhere in the instructions, where appropriate.

There were no practice trials. When the participants indicated that they understood the instructions, they were shown how to proceed through the stimuli by moving the cursor to a box saying “show next movie” and clicking on it. The experimenter monitored the participants as they ran through this procedure.

Stimuli were randomly ordered and a different random order was generated for each participant. At the end of the session, participants were thanked and given course credit and a debriefing sheet, which informed them about the research topic but did not mention the specific hypotheses being tested.

All procedures performed in this study were in accordance with the ethical standards of the author’s institution and were approved by the institution’s Ethics Committee, and with the 1964 Helsinki declaration and its later amendments or comparable ethical standards.

## Results

Data were initially analysed with a 2 between (kind of phenomenal causality, launching v. entraining) × 2 between (common fate object, causal object v. target) × 2 within (target prior motion, stationary v. right-to-left) × 5 within (number and location of common fate objects, 4 v. 2 at positions 2 and 3—hereafter 2/2&3—v. 2/3&4 v. 1/3 v. 1/4) × 2 within (spatial separation of common fate objects, narrow v. wide) mixed design analysis of variance (ANOVA). Effect sizes for significant effects were computed using partial eta squared (Fritz et al. 2012).

There was no significant main effect of kind of phenomenal causality, *F* (1, 116) = 0.02, MSE = 9133.10, *p* > 0.05. The only significant effect involving this factor was an interaction with target prior motion which will be described shortly. There was no significant effect of common fate object, *F* (1, 116) = 0.74, MSE = 9133.10, *p* > 0.05. There was just one significant effect involving this factor, a three-way interaction with number and location of targets and spatial separation, which also will be described shortly.

There was a significant effect of number and location of common fate objects, *F* (4, 464) = 57.66, MSE = 424.50, *p* < 0.001, *η* ^2^_*p*_ = 0.33. Post hoc paired comparisons with the Tukey’s test revealed that the mean for four objects (73.18) was significantly higher than all of the others. In addition, the mean for one object at location 4 (57.12) was significantly lower than all others. The mean for two objects in locations 2 and 3 (69.96) was significantly higher than those for one object at location 3 (65.74) and one object at location 4 (57.12), but was not significantly higher than that for two objects in locations 3 and 4 (67.04). The result for four objects supports the prediction, and the tendency for means for stimuli with two objects to be higher than means for stimuli with one object also does so. This main effect was qualified by two significant interactions. Neither showed any trends that violated the effects just described; they will be described further shortly.

There was a significant effect of spatial separation, *F* (1, 116) = 35.11, MSE = 405.72, *p* < 0.001, *η* ^2^_*p*_ = 0.23, with a higher mean at narrow separation (69.04) than at wide separation (64.17). This supports the prediction.

There was a significant effect of target prior motion, *F* (1, 116) = 91.29, MSE = 556.59, *p* < 0.001, *η* ^2^_*p*_ = 0.44 with a higher mean with right to left prior motion (71.21) than with the objects stationary (62.01). This supports the prediction.

There was, as mentioned earlier, a significant interaction between target prior motion and kind of phenomenal causality, *F* (1, 116) = 14.58, MSE = 556.59, *p* < 0.001, *η* ^2^_*p*_ = 0.11. Simple effects analysis revealed no significant effect of kind of phenomenal causality. The effect of target prior motion, however, appeared to be stronger with launching stimuli, *F* (1, 116) = 89.42, MSE = 556.59, *p* < 0.001, *η* ^2^_*p*_ = 0.44, than with entraining stimuli, *F* (1, 116) = 16.45, MSE = 556.59, *p* < 0.001, *η* ^2^_*p*_ = 0.12. In both cases, the difference was in the same direction as the main effect. This did not interact significantly with the common fate object factor, which means that it made no significant difference whether the target was one object or multiple objects, so this result does not seem to bear on the hypotheses about common fate.

There was also a significant interaction between target prior motion and number and location of common fate objects, *F* (4, 464) = 3.95, MSE = 127.30, *p* < 0.01, *η* ^2^_*p*_ = 0.03. Simple effects analyses revealed that all comparisons were statistically significant in the direction of the respective main effects. It seems that the effect of number and location of common fate objects was stronger when the target was initially stationary, *F* (4, 464) = 52.02, MSE = 226.37, *p* < 0.001, *η* ^2^_*p*_ = 0.31, than when the target initially moved from right to left, *F* (4, 464) = 30.31, MSE = 203.31, *p* < 0.001, *η* ^2^_*p*_ = 0.21, but both effects were strongly significant. It is possible that establishment of common fate by launching or entraining is stronger than establishment of common fate by prior motion, but the trends in respect of the hypotheses were similar under both conditions.

There was just one other significant effect, the aforementioned three-way interaction between common fate object, number and location of common fate objects, and spatial separation, *F* (4, 464) = 3.55, MSE = 168.15, *p* < 0.01. *η* ^2^_*p*_ = 0.03. Means conformed to the directions of the main effects. However, for the condition with two common fate objects in positions 2 and 3, there was a strong effect of spatial separation when the common fate objects were the targets but no significant effect when the common fate objects were the causal objects. This does not seem to have any implications for the hypotheses.

## Discussion

The main hypotheses about common fate were supported. Reported impressions of both launching and entraining were stronger when the common fate objects were four objects than when they were fewer, and also stronger when they were two than when there was just one. This tendency was not significantly affected by whether a launching or an entraining stimulus was used, nor by whether the common fate objects were the causal object or the target. In addition, as predicted on the basis of findings reported by Uttal et al. (2000) and Stürzel and Spillmann (2004), the causal impression was stronger when the spatial separation of the common fate objects was narrow than when it was wide, except for the condition with two common fate objects as causal objects in positions 2 and 3, where there was no statistically significant effect. Also as predicted, the causal impression was stronger when the target initially moved from right to left than when it was stationary. However, whether the target or the causal object was the common fate object made no significant difference to this tendency. It is therefore likely that it reflects a difference in perceived force and not an effect of common fate.

Although the manipulation of number and location of common fate objects did not interact significantly with whether the stimulus was launching or entraining, this should not be taken as indicating that there are no differences in the factors that affect the occurrence and strength of the launching and entraining impressions. The effect of target prior motion was stronger with launching than with entraining stimuli, though significant with both. It is not clear how this should be interpreted. The critical difference between launching and entraining is that launching is a momentary event where the causal relation is confined to the time of contact between the objects, whereas in entraining, the causal object is perceived as acting on the target object for an extended period of time because of the temporally extended contact between them. Possibly prior motion of the target object is perceived as influential at time of contact but not after that. This would entail a reduced effect of target prior motion with entraining stimuli because the action of the causal object on the target object is not perceived as influenced by the target’s prior motion after the initial contact between them has occurred. Further investigation of this could shed more light on the differences between the launching and entraining impressions, which have hitherto been the subject of hardly any direct comparisons (Hubbard 2013a, b, c; Michotte 1963).

The reported causal impressions were stronger when there were four objects than when there were two. One possible explanation of this is that there could be differences in the implied mass of the common fate object: the common fate object might be perceived as more massive when it encompassed four visible objects than when it encompassed two, because of the greater amount of space it occupied. Size is not necessarily a valid guide to mass: a balloon is lighter than a golf ball, for example. It is possible that size is a visual cue to mass in the absence of other information (Kotovsky and Baillargeon 1998). That would, however imply a prediction that the causal impressions should be stronger when the spatial separation of the objects was wider than when it was narrow, because wide separation creates a larger perceptual object than narrow separation does. That prediction is disconfirmed by the present results that show a significant difference in the opposite direction. It is, therefore, unlikely that differences in perceived mass account for the effect of four versus two objects.

## Experiment 2

Common fate is determined by kinematic properties: objects that share common motion properties (e.g. velocity, time of onset) tend to be grouped together. This proposition yields two hypotheses that were tested in Experiment 2. One is that common fate does not occur for objects that differ in their motion properties, so the causal impression should be weaker for stimuli in which the objects have different motion properties than for stimuli in which the objects have the same motion properties. The other is that the spatial relations between the objects do not contribute to common fate: the objects can be arranged in any way, so long as they share the same motion properties. In Experiment 1, the objects were vertically aligned and moved horizontally. In Experiment 2 the vertical alignment is disrupted to varying degrees, and it is predicted that this will not significantly affect the causal impression. There is an important qualification to this. Note first that the stimuli used in Experiment 2 were launching stimuli with a single causal object and four target objects, and the argument is expressed with reference to that kind of stimulus. The qualification is that the causal object should stop adjacent to a contour of the perceived object (i.e. the object that encompasses all of the target objects); more precisely, that this should be controlled across the manipulation of the spatial array. If this is not the case, then the causal impression may be weakened by a factor not connected to common fate, namely the presence of a spatial gap between the stopping point of the causal object and the boundary of the perceived target object (Michotte 1963; Yela 1952). Stimuli for Experiment 2 were devised with this qualification in mind.

## Method

Details of method were as for Experiment 1 with the following differences. There were 30 participants, none of whom had participated in Experiment 1. Stimuli consisted of three sets of manipulations and one “standard” stimulus used for hypothesis testing purposes. There were 27 stimuli in all, comprising the standard stimulus, 6 in set 1, 12 in set 2, and 8 in set 3. All stimuli were presented to each participant, and order of presentation was randomised independently for each participant. The manipulations are summarised in Table 1 and will now be described in detail.

### Standard stimulus

The standard stimulus was a launching stimulus resembling that depicted in Fig. 1, with a single causal object and four target objects. The critical features of the standard stimulus were that the four target objects in their initial locations were vertically aligned, that they all started to move at the same moment, and that they all moved with the same speed. These features were manipulated in three sets of stimuli described below. The experiment was designed, in part, to test whether the causal impression was weaker under the experimental manipulations described below than with this standard stimulus. Thus, statistical analyses included comparisons between the standard stimulus and individual stimuli in each set.

### Vertical offset set

The stimuli in this set were the same as the standard stimulus, except that the initial positions of the target objects were horizontally offset by varying amounts. In each stimulus, one object was not offset, so as to maximise the proximity of the perceived object boundary to the point at which the causal object stopped moving. Successive objects were then displaced by equal amounts. Three different total amounts of displacement were used, namely 1.05, 2.10, and 3.15 cm. The order in which objects were displaced was manipulated. One order, from zero to maximum displacement, was object 2, 4, 1, and 3, and the other was the opposite of that. These manipulations created a total of 6 stimuli. All other features of the stimuli were as in the standard stimulus. Since the objects share the same motion properties throughout the stimulus, they possess common fate despite the offset initial locations, so it is predicted that there will be no significant effect of the spatial displacement manipulation.

### Motion onset delay set

The stimuli in this set were the same as the standard stimulus, except that delays in motion onset of individual target objects were introduced, with two independent variables. Delay before the first object started moving was either zero or 54 ms. Delay between onset of motion in the first and last target objects to move was either 162, 324, or 486 ms. The order in which the objects started to move was manipulated with the same two orders as for the spatial displacement manipulation in the vertical offset set. These manipulations created a total of 12 stimuli. All other features of the stimuli were as in the standard stimulus. Having the objects start to move at different times should rule out common fate, although this could be mitigated by the fact that they all end up with common motion properties. It is predicted that the delay manipulations will significantly weaken the causal impression by comparison with the standard stimulus.

### Speed variation set

The stimuli in this set were the same as the standard stimulus, except that the target objects moved at different speeds. Two amounts of variation in speed were used, one with a difference of 8.1 cm/s between the slowest object and the fastest, and one with a difference of 16.2 cm/s. Two speeds of the slowest object were used, 2.7 and 8.1 cm/s. The order of objects with different speeds from slowest to fastest was manipulated with the same two orders as for the spatial displacement manipulation in the vertical offset set. These manipulations created a total of 8 stimuli. All other features of the stimuli were as in the standard stimulus. Motion at different speeds means that there is no common fate, so it is predicted that both speed manipulations will significantly weaken the causal impression by comparison with the standard stimulus.

## Results

### Vertical offset set

For purposes of comparison with the standard stimulus, data were initially analysed with a one-way ANOVA with 7 levels, comprising the standard stimulus and the 6 vertical offset stimuli. The result was not significant, *F* (6, 174) = 1.73, MSE = 142.39, *p* > 0.05. A planned two-way ANOVA was also carried out on the six vertical offset stimuli, and again no significant results were found. For total amount of displacement, *F* (2, 58) = 1.29, MSE = 160.16, *p* > 0.05. For order, *F* (1, 29) = 0.00, MSE = 121.58 *p* > 0.05. For the interaction, *F* (2, 58) = 0.96, MSE = 97.76, *p* > 0.05. Means ranged from 59.50 to 68.83. These results are consistent with the prediction.

### Motion onset delay set

For purposes of comparison with the standard stimulus, data were initially analysed with a one-way ANOVA with 13 levels, comprising the standard stimulus and the 12 motion onset delay stimuli. This yielded a significant result, *F* (12, 348) = 20.39, MSE = 239.82, *p* < .001, *η* ^2^_*p*_ = 0.41. Post hoc paired comparisons with the Tukey’s test revealed that the standard stimulus received higher ratings than all other stimuli.

A planned 2 (first object delay, 0 v. 54 ms) × 3 (total motion onset delay, 162 v. 324 v. 486 ms) × 2 (object motion onset order, 2/4/1/3 v. 3/1/4/2) ANOVA was carried out. Means are shown in Table 2. For comparison with those means, the mean for the standard stimulus was 68.83.

**Table 2:** Mean causal impression ratings, Experiment 2, motion onset delay set

Total delay	Object order	First object delay	
		0 ms	54 ms
162 ms	2/4/1/3	50.40	40.20
	3/1/4/2	44.47	46.17
324 ms	2/4/1/3	34.07	25.90
	3/1/4/2	34.87	30.80
486 ms	2/4/1/3	28.77	29.13
	3/1/4/2	26.10	23.20

There was a significant effect of total motion onset delay, *F* (2, 58) = 33.06, MSE = 336.98, *p* < 0.001, *η* ^2^_*p*_ = 0.53. Post hoc paired comparisons with the Tukey’s test revealed a significantly higher mean for 162 ms than for 324 and 486 ms, which did not differ significantly. The effect of first object delay fell short of statistical significance, *F* (1, 29) = 3.62, MSE = 374.12, *p* > 0.05.

There was just one other significant result, an interaction between all three variables, *F* (2, 58) = 3.27, MSE = 132.00, *p* < 0.05, *η* ^2^_*p*_ = 0.10. As can be seen in Table 2, in two cases the mean for zero delay was higher than that for 54 ms delay. These were 162 ms total delay and for 324 ms delay, both with the 2/4/1/3 order. In the other four cases there was no significant difference. There is no readily apparent explanation for this interaction; it may be just that there is a weak effect of first object delay that happens to reach statistical significance in some comparisons and not in others.

### Speed variation set

For purposes of comparison with the standard stimulus, data were initially analysed with a one-way ANOVA with 9 levels, comprising the standard stimulus and the 8 speed variation stimuli. This yielded a significant result, *F* (8, 232) = 11.21, MSE = 322.61, *p* < 0.001, *η* ^2^_*p*_ = 0.28. The standard stimulus had a mean rating of 68.83 which was higher than that of any other stimuli in this set, and post hoc paired comparisons revealed that it was significantly higher in 4 out of 8 comparisons (*p* < 0.05).

A planned 2 (range of speeds, 8.1 v. 16.2 cm/s) × 2 (slowest object speed used, 2.7 v. 8.1 cm/s) × 2 (object motion onset order, 2/4/1/3 v. 3/1/4/2) ANOVA was carried out. Means are shown in Table 3.

**Table 3:** Mean causal impression ratings, Experiment 2, speed variation set

Range of speeds	Object order	Slowest object speed	
		2.7 cm/s	8.1 cm/s
8.1 cm/s	2/4/1/3	40.63	54.27
	3/1/4/2	37.60	57.13
16.2 cm/s	2/4/1/3	40.63	58.47
	3/1/4/2	41.90	59.07

Just one effect was significant, a main effect of slowest object speed used, *F* (1, 29) = 29.68, MSE = 587.10, *p* < .001, *η* ^2^_*p*_ = 0.51, with a higher mean at 8.1 cm/s (57.23) than at 2.7 cm/s (40.19).

## Discussion

The results for the vertical offset and motion onset delay sets were in accordance with the predictions. There was no significant effect of manipulating the spatial aligment of the objects in stimuli in which they all shared the same kinematic properties (vertical offset set). This indicates both that sharing kinematic properties is sufficient for binding them into a single perceived object and that a strong causal impression occurs with a launching-type stimulus involving such an object as the target. Staggering the time of motion onset across the four objects (motion onset delay set) resulted in substantially reduced causal impressions, consistent with the reasoning that the temporal manipulation would disrupt common fate, and thereby preclude perception of a single object encompassing the four visible objects, with consequent weakening of the causal impression.

The standard stimulus received a higher mean rating than any of the stimuli in the speed variation set, but the difference was significant in only 4 out of 8 comparisons. This indicates that having the objects start to move at the same time but with different speeds is not as disruptive to the causal impression as the reasoning based on common fate would predict. A close look at the results suggests a possible explanation for this. There was an effect of only one of the manipulated variables, the slowest object speed used, with higher ratings at the faster speed (8.1 cm/s) than at the slower speed (2.7 cm/s). The noteworthy feature of this is that the stimuli concerned differ in other ways, but those differences seem not to have affected the causal impression. For example, when the slowest object moved at 8.1 cm/s, the range of speeds was from 8.1 to 16.2 cm/s in the narrow range of speeds, but from 8.1 to 24.3 cm/s in the wide range of speeds, and yet this difference in the range of speeds had no significant effect. This indicates that the causal impression was affected only by the slowest moving object. In other words, ratings were moderately high (range 54.27–59.07) not because the launcher was perceived as making all the four objects move but at different speeds, but because it was perceived as making only the slowest object move. This is the only way to account for the lack of effect of the range variable. It is perhaps relevant that the means, ranging from 54.27 to 59.07, were similar to the means for the one-object stimuli in Experiment 1 (65.74 and 57.12). Thus, the manipulation has indeed disrupted the causal impression associated with the perceived object constructed by common fate, but the resultant causal impression is not much reduced because the manipulation results in an impression that the launcher makes just one of the four target objects move. Why should that one be the slowest of the four? The launching impression is weak or absent when the target object moves faster than the causal object, and tends to be replaced by an impression called “triggering” (Michotte 1963), in which the target object's motion as perceived as its own autonomous motion, but initiated by contact from the causal object (Hubbard 2013a; Michotte 1963; Natsoulas 1961). It is likely, therefore, that the launching impression occurred only for the slowest object of the four, and that triggering impressions occurred for the other objects, which were mainly perceived as moving of their own accord.

## General discussion

In two experiments, launching and entraining stimuli were presented in which either the launcher or the target consisted of four spatially separated objects that shared identical motion onset time and velocity, and the other object was a single object, as is customary with such stimuli. There was no visible contact between the single object and any of the four other objects. Strong causal impressions were reported, that were not significantly affected by whether a launching or an entraining stimulus was used, nor by whether the four objects were the target and the single object was the launcher or vice versa. The reported causal impression was significantly reduced, in Experiment 1, by manipulations known to reduce or eliminate common fate: the causal impression was weaker if there were two objects or one instead of four; the causal impression was weaker with greater spatial separation of the four objects; and when the four objects were the target, the causal impression was stronger if the four objects were initially in motion with identical motion properties than if they were stationary. In Experiment 2, the causal impression was not disrupted by a change to the spatial alignment of the objects. The causal impression was, however, disrupted by changes to the kinematics such that different objects moved in different ways, starting to move at different times but with the same speed, or moving at different speeds. The change in kinematic properties meant that there was no common fate, but common fate was preserved with the change in spatial alignment alone. Overall, if the motions of the four objects had common fate, the causal impression was stronger than would normally be the case, given that there was a spatial gap between the launcher and the target (Michotte 1963; Yela 1952).

An important implication of this set of findings is that the perceptual interpretation of visual stimuli in phenomenal causality research may include specification of objects present in spaces where no object is visible. In the present case, the spaces between the four objects that share common motion properties are interpreted as unseen parts of a single object, of which the four visible objects are also part. The visual system is presumably adapted to a complex visual environment in which moving objects are sometimes partly occluded (e.g. by branches of trees or by railings), and is able to make a perceptual construct of a single object despite the partial occlusions. The same reasoning applies to other instances of perceptual impressions of causality. For example, White and Milne (1997) presented a stimulus in which five objects were arranged, initially stationary, in a columnar formation with gaps between them. First the topmost object began to move, then the second object started to move with the same motion properties, and so on until all five objects were in motion. Observers consistently reported a strong impression that the first object was pulling the others. Strictly speaking, this is impossible because there was no visible connection between the objects. However, by the likelihood principle, it is very unlikely that the five objects would all share the same motion properties (except for time of motion onset) by chance. It is more likely that the first object is indeed pulling the others, and that there is a physical connection between them that is not visible. The gap stimuli used by Michotte (1963) and Yela (1952) can be interpreted in the same way. A launcher cannot make a target move if there is an unbridged gap between them, and the temporal coincidence between the launcher stopping and the target starting to move is unlikely to occur by chance. However, the temporal properties of the stimulus are not so unlikely if there is a stationary object in the gap, contacting the target and contacted by the launcher, through which the momentum of the launcher is transferred to the target. Again, the object in the gap is not visible, but its presence forms part of the perceptual interpretation of the stimulus. These examples show that the causal impression is integrated into a complex, coherent perceptual interpretation of the stimuli used in phenomenal causality research.

The reasoning about the effect of manipulations of common fate on the perceptual impression of causality was guided by the likelihood principle. It is certainly plausible in this case, on the grounds that the distal stimulus for a set of four objects whose motion exhibits common fate is more likely to be a single object under imperfect viewing conditions than four separate objects that happen to share motion properties. However, it is notoriously difficult to distinguish the likelihood principle from the simplicity principle, the principle that the percept constructed is the simplest interpretation of the stimulus information (Wagemans et al. 2012). Other explanations for Gestalt perceptual phenomena have also been proposed though apparently not yet explicitly applied to common fate (Wagemans et al. 2012). It remains possible that some explanatory principle other than likelihood would be the correct explanation for the results reported here; hopefully ways of distinguishing between the alternative principles can be devised.

In the stimuli where the target is the common fate object, the effect of common fate on the causal impression must be retrospective; that is, motion of the four objects after contact influences the percept of the contact event itself. This is not as odd as it might sound: the causal impression cannot occur until some time after contact because information about the target’s motion after contact is required. Michotte (1963) reported that no causal impression occurs if the target does not move at or after contact, and the kind of causal impression that does occur depends, in part, on the relative speeds of the two objects (Michotte 1963; Natsoulas 1961). Choi and Scholl (2006) showed that perceptual interpretation of a stimulus as launching or as a kind of event not involving causality can be influenced by another event that does not occur until about 200 ms after the target in the first stimulus has started to move. So, insofar as it is an interpretation of the contact event, the causal impression is inevitably a postdictive phenomenon (Shimojo 2014).

As is always the case in experiments on phenomenal causality, there is a danger that participants’ responses could be influenced by post-perceptual processing. Of particular relevance in this case is the issue of demand characteristics, which are cues in the experimental situation that might convey the hypotheses and predictions to participants, who are then motivated to produce the apparently desired behaviour (Orne 1962). The instructions stated that “people often do have a visual impression that one object made one or more others move even under conditions where this could not happen in real life” (p. 12); this was intended to introduce participants to the possibility that causal impressions could occur even when moving objects do not come into contact. This might lead participants to report causal impressions they did not have. However, the issue is whether this reasoning could predict the specific pattern of results obtained here. One might expect a general inflation of ratings, if this statement infuenced responses in the manner of demand characteristics. However, the statement does not provide any basis for differentiating between the stimuli. Mere exposure to a variety of stimuli does not provide any cues as to which ones should receive higher ratings. Participants could only generate the observed patterns of responses in the absence of causal impressions if they were familiar with the research literature on common fate, such as the studies by Uttal et al. (2000) and Stürzel and Spillmann (2004) that were the basis of the predictions for Experiment 1, and if they could apply that knowledge to the conditions of the experiment. This seems unlikely, so it is unlikely that the tests of the predictions were compromised by the wording of the instructions.

In conclusion, whether or not Michotte’s (1963) Gestalt interpretation of the launching and entraining effects is correct, the occurrence and strength of both causal impressions can be influenced by common fate, such that the causal impression can occur when the launcher stops in empty space, if the space adjacent to its stopping location is perceptually interpreted as the unseen boundary of an object defined by the common fate of the motions of visible objects around it.

## Footnotes

1 The locations of the two-object and one-object stimuli were originally chosen to test another aspect of the common fate hypothesis. For the two-object stimuli, the objects in locations 2 and 3 are either side of the plane of motion of the other object (the launcher in the Fig. 1 stimulus). This is necessary to control symmetricality of the arrangement in the comparison with the four-object stimulus. The objects in locations 3 and 4 are both below the plane of motion of the other object. In this case, it might seem as though the common fate object does not extend as far up as the plane of motion of the other object, and a correspondingly weaker causal impression might be expected. On the other hand, the fact that these two objects both start to move with shared motion properties at the moment the other objects stops might be sufficient, as an unusual coincidence, to prompt a phenomenal extension of the common fate object up at least as far as the plane of the other object’s motion, so that the impression would be that the other object struck that phenomenal extension and thereby set the two visible parts of the common fate object in motion. The lack of significant difference between the rated causal impression for the two two-object stimuli is consistent with the latter possibility and not with the former, but is not conclusive. The finding that the mean rating for the one object at location 4 was significantly lower than that for all other conditions, including the one object at location 3, makes interpretation of the results more problematic, because it suggests a simple effect of gap size (the object in location 4 is further from the stopping point of the causal object than the object in location 3 is), and that makes it possible that the result for the two-object stimuli is confounded by an effect of gap size as well. The issue of the comparisons between these stimuli and their possible interpretations is demoted from the main text for the sake of clarity.
